# Flexible and ultra-lightweight polymer membrane lasers

**DOI:** 10.1038/s41467-018-03874-w

**Published:** 2018-05-01

**Authors:** Markus Karl, James M. E. Glackin, Marcel Schubert, Nils M. Kronenberg, Graham A. Turnbull, Ifor D. W. Samuel, Malte C. Gather

**Affiliations:** Organic Semiconductor Centre, SUPA, School of Physics and Astronomy, University of St Andrews, St Andrews, KY16 9SS UK

## Abstract

Organic semiconductors enable the fabrication of a range of lightweight and mechanically flexible optoelectronic devices. Most organic semiconductor lasers, however, have remained rigid until now, predominantly due to the need for a support substrate. Here, we use a simple fabrication process to make membrane-based, substrate-less and extremely thin (<500 nm) organic distributed feedback lasers that offer ultralow-weight (*m*/*A<*0.5 gm^−2^) and excellent mechanical flexibility. We show operation of the lasers as free-standing membranes and transfer them onto other substrates, e.g. a banknote, where the unique lasing spectrum is readily read out and used as security feature. The pump thresholds and emission intensity of our membrane lasers are well within the permissible exposures for ocular safety and we demonstrate integration on contact lenses as wearable security tags.

## Abstract (web summary)

Organic semiconductors provide a platform for flexible lasers, but these are still produced on rigid, thick substrates. Here, Karl et al. develop a method to make freestanding membrane lasers that can be transferred onto any substrate and show that these could be used as anti-counterfeiting labels.

## Introduction

Optically pumped organic solid-state lasers have gained widespread attention as coherent light sources that are easy to fabricate, have emission tunable across the whole visible range, and are potentially disposable and biocompatible^1–8^. These lasers hold great promise for a number of applications, e.g. for on-chip spectroscopy^9,10^, data-communication^11^, biosensing^12^, and chemosensing for detecting explosives^13,14^. However, while organic LEDs, solar cells, and field-effect transistors are now routinely made in bendable or even stretchable formats and with extremely low specific weights^15–19^, most organic lasers have remained rigid and relatively bulky, largely due to a need for macroscopic and solid support substrates (typical substrate thickness, >100 µm). Many organic lasers use distributed feedback (DFB) resonators, which provide strong in-plane optical feedback^20^. There have also been examples of flexible DFB designs^21–25^. However, these use substrates or matrices of macroscopic thickness, or require metal oxide intermediate layers and femtosecond pumping schemes. These requirements have limited applications of flexible DFB lasers so far.

Here, we introduce a different organic laser that maximizes mechanical flexibility and reduces the thickness of the laser to its ultimate limit, by using an architecture that comprises only the organic semiconductor and a DFB resonator and that is fabricated by a wholly solution-based process. The resulting 200-nm thick membrane lasers were operated freestanding in air or readily transferred onto a new substrate, on which direct fabrication of a laser may otherwise be impossible or impractical. As an example, we show how membrane lasers that were designed to produce a well-defined and unique lasing spectrum can be used as counterfeit-resilient, barcode-type security labels on bank notes. In another example, a laser beam was emitted from a bovine eye onto which a contact lens with a membrane laser had been mounted. Due to the low threshold of our membrane laser, a similar configuration is expected to be safe to use in the human eye, e.g. to complement biometric iris recognition.

## Results

### Membrane laser design and fabrication

**Fig. 1:**
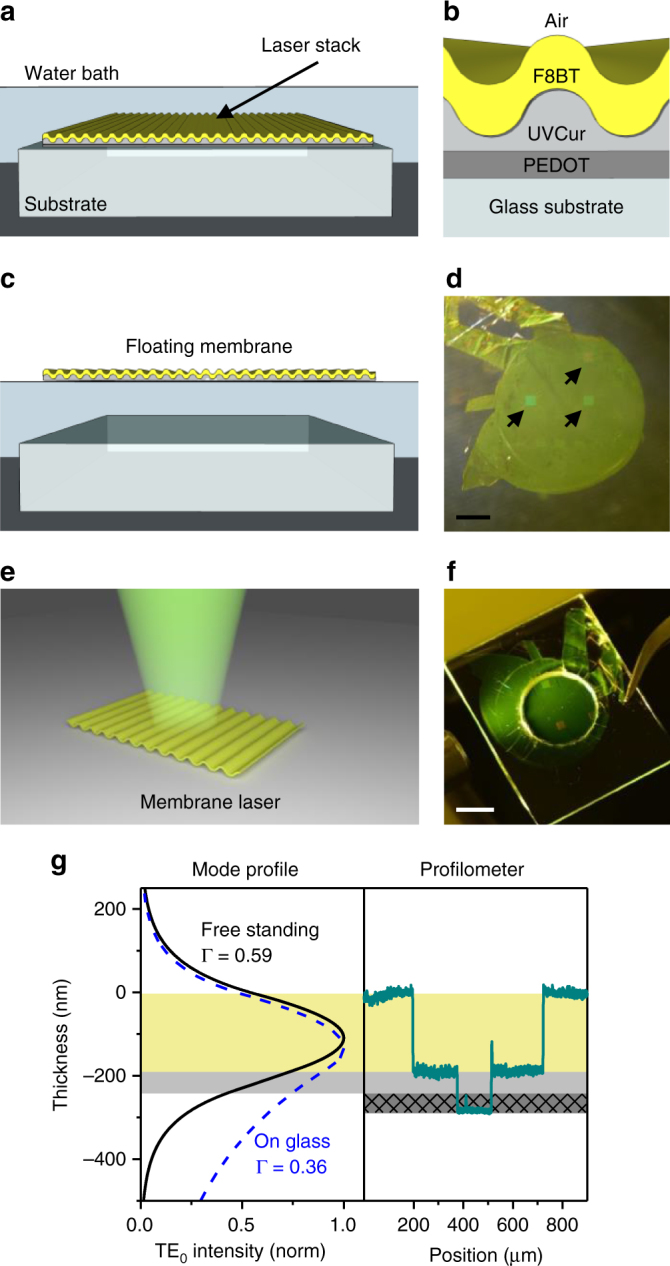
Fabrication and physical properties of the membrane laser. **a** Schematic of the laser stack immersed in water. **b** Composition of the laser stack before lift-off (not to scale) consisting of a glass substrate, the sacrificial layer (PEDOT), the polymer grating (UVCur), and the polymer gain material (F8BT). **c** Schematic of a floating membrane post lift-off. **d** Image of a floating membrane post lift-off. Black arrows indicating the position of three second-order distributed feedback (DFB) gratings. Scale bar, 3 mm. **e** Schematic of the vertical laser emission from a second-order DFB laser membrane (pump spot not shown). **f** Image of a free-standing membrane laser suspended over a hole in a glass substrate. Scale bar, 5 mm. **g** Mode profile of the TE_0_ mode intensity ($\left| {E_y} \right|^2$) in a free-standing membrane laser (black solid line) and a conventional laser on a glass substrate (blue dashed line). Profilometer measurement of the membrane before lift-off (green). The yellow area indicates the gain layer, the light gray area the residual grating layer and the patterned dark gray area the sacrificial layer. Γ quantifies the overlap of the TE_0_ mode with the gain material

To produce transferable and thin membrane lasers, we developed a water-based lift-off technique that releases the final device from a carrier substrate at the end of the fabrication process (Fig. 1a). The laser was produced via solution-based deposition and UV nanoimprint lithography. The stack initially consisted of a thick and rigid carrier glass substrate, a ~50-nm thick water soluble sacrificial layer of (poly(3,4-ethylenedioxythiophene)-polystyrene-sulfonate (PEDOT:PSS), a UV curable imprint resist defining the DFB resonator and a (180 ± 10)-nm thick layer of an organic semiconducting polymer (e.g. F8_0.9_BT_0.1_, Methods) as gain material (Fig. 1b). When immersing this stack in water, a hydrophobic membrane detached from the substrate and spread out on the water surface (Fig. 1c, d). The membrane was readily picked up, and then either suspended in air or transferred onto another substrate (Fig. 1e, f). The lift-off procedure had no detrimental effect on the photoluminescence quantum yield (PLQY) of the organic polymer (Supplementary Table 1). We applied the above technique to fabricate membrane lasers with different periodicities and gain materials based on one-dimensional second-order and mixed-order DFB gratings^26^ and obtained groove depths of (106 ± 5) nm and (86 ± 4) nm, respectively (Supplementary Fig. 1).

Removing the substrate not only rendered the laser flexible and lightweight; replacing the glass substrate (refractive index, *n* = 1.52) by air also improved the confinement of the lasing mode to the gain material (*n* = 1.7) and reduced spontaneous emission losses by eliminating leaky substrate modes. The mode overlap with the gain material improved from Γ = 0.36 for a typical laser stack on a glass substrate to Γ = 0.59 for our membrane design (Fig. 1g).

### Membrane laser emission

**Fig. 2:**
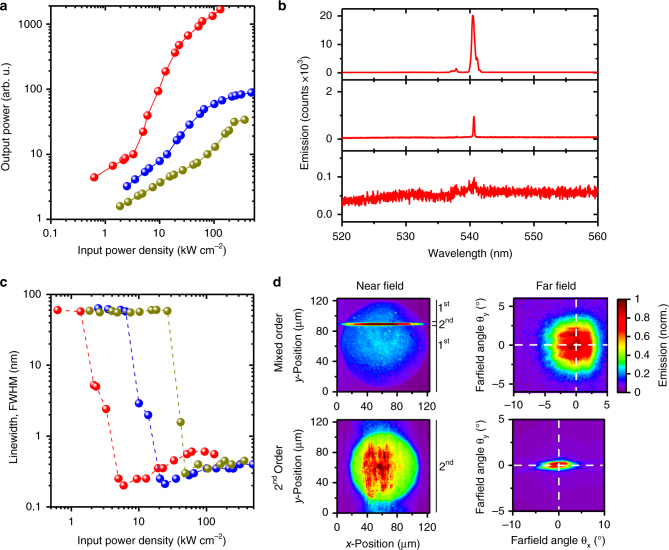
Characterization of membrane lasers. **a** Input−output characteristics for mixed-order membrane lasers with different gain materials. The lasing thresholds for F8_0.9_BT_0.1_ (red), F8BT (blue) and Super Yellow (yellow)-based devices are 3.3 kW cm^−2^, 13.8 kW cm^−2^ and 75.6 kW cm^−2^, respectively. **b** Emission spectra of the F8_0.9_BT_0.1_-based membrane laser for pump fluences below (2.3 kW cm^−2^), around (5.0 kW cm^−2^) and well above (33 kW cm^−2^) the lasing threshold. **c** Spectral linewidth (full width at half maximum, FWHM) vs. input-power density for the devices based on F8_0.9_BT_0.1_ (red), F8BT (blue) and Super Yellow (yellow). **d** Near and far field emission from a mixed- and a second-order distributed feedback (DFB) membrane laser. The location of regions with first- and second-order grating period is indicated on the right-hand side of the near field emission

Devices based on mixed-order gratings and F8_0.9_BT_0.1_ as the gain material showed laser emission under pulsed excitation (excitation wavelength, *λ* = 450 nm; pulse duration, 5 ns) with a threshold pump fluence of 3.3 kW cm^−2^ (Fig. 2a and Supplementary Fig. 2), in line with state-of-the-art organic DFB lasers^27,28^. Figure 2b shows emission spectra of the same membrane laser and Fig. 2c summarizes the evolution of spectral line width with pump fluence. At low pump fluences the spectrum was dominated by the fluorescence background (Δ*λ* ≈ 60 nm) with a broad Bragg mode (Δ*λ* ≈ 2–4 nm) at a wavelength of *λ* ≈ 540 nm. Above threshold, a single lasing mode (Δ*λ* ≈ 0.2–0.5 nm) appeared and gained superlinearly in relative intensity, eventually completely dominating the emission spectrum. Well above threshold, our second- and mixed-order membrane lasers showed emission linewidths of (133 ± 14) pm and (498 ± 50) pm, respectively (full width at half maximum, Supplementary Fig. 3). We also investigated the near and far field emission of mixed- and second-order DFB grating membrane lasers (Fig. 2d). The near field emission data for the mixed-order grating indicate that most laser light was coupled out from the narrow second-order region in the center of the grating structure^26^. Due to the narrow width of this region, the emitted laser beam was rather divergent (spread of far field emission at half maximum, ±(2.60 ± 0.04)°). However, if necessary, the divergence can be reduced by adjusting the number of light-extracting second-order periods inserted between the first-order period feedback structure^29^. For the pure second-order DFB membrane gratings, the light extraction was enhanced and spread over a larger area as can be seen in the near field emission pattern. This led to higher lasing thresholds (typically, >60 kW cm^−2^), but to reduced beam divergence (spread, ±(0.34 ± 0.04)°) and a sharp far field emission pattern with a fine double-lobe structure^30^. Well-defined far field emission and low divergence are clear indications of spatial coherence and further evidence for laser action in our membranes^2^.

The process for laser membrane fabrication is compatible with a range of conjugated polymers of different chemical structure and molecular weight. So far, we tested F8_0.9_BT_0.1_, F8BT and Super Yellow (SY) and found F8_0.9_BT_0.1_ to provide the best laser performance (i.e., lowest threshold) among these, in line with earlier findings on rigid substrates and with the PLQY of these materials^31,32^ (Supplementary Table 1).

### Membrane lasers as security features on banknotes

**Fig. 3:**
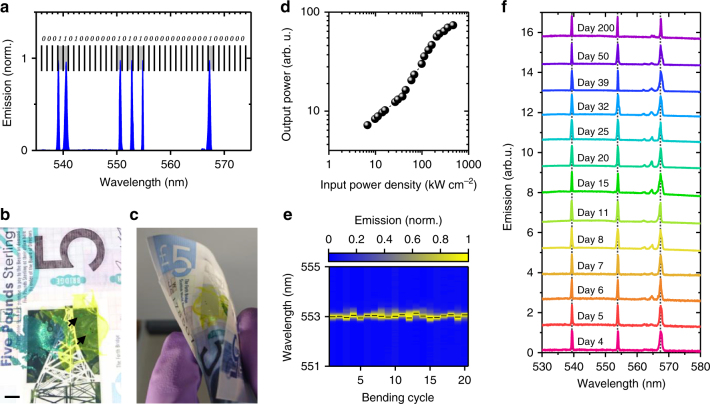
Membrane lasers as security features on banknotes. **a** Barcode-like narrow-band lasing emission from a combination of different second-order gratings with periods ranging from 340 to 360 nm. **b** Photograph of a £5 banknote with a series of membrane lasers transferred on the transparent window of the banknote. Two second-order distributed feedback (DFB) gratings are indicated by black arrows. Scale bar, 5 mm. **c** Photograph of same banknote when bent parallel to the grating grooves. **d** Input−output characteristics of a mixed-order DFB grating membrane laser on a banknote. **e** Lasing spectra of a banknote with a second-order membrane laser after repeated bending (radius of curvature, ~ 8 mm). Black dashes mark the measured peak wavelength after each bending cycle. **f** Lasing spectra acquired from a banknote containing a membrane with three different gratings over a time span of 200 days. For clarity, the spectra are offset vertically

Two important features of our membrane lasers are their transferability and mechanical flexibility. After lift-off, the membranes can be transferred onto a wide range of substrates, independent of the substrate composition and surface topology. After the water used for the lift-off has evaporated, the membranes stick tightly to the new surface. This enables the use of membrane lasers as novel barcode-like security labels for objects requiring authenticity control (e.g. banknotes, ID documents, etc.). The emission spectrum of membrane lasers can be tuned by the grating period, the choice of gain material and the waveguide design. The distinct single-mode emission can either be used as an identification feature on its own or be further enhanced by combining a number of different gratings on a single membrane. This creates a well-defined and discrete lasing spectrum that resembles a binary barcode, which is unique to each membrane laser and which can be read out rapidly (ns pumping) and without physical contact (Fig. 3a). If the laser lines generated by different gratings are spaced by 1 nm (which appears feasible, see long-term measurement below) and cover the >50 nm wide gain spectrum of the organic polymer, at least 50 independent spectral channels can be encoded. This translates to about 2^50^ ≈ 10^15^ unique labels.

In the following, we illustrate the application of this concept as a security feature on banknotes. Figure 3b, c shows a polymer banknote with a membrane laser transferred onto the transparent window of the banknote. One of the reasons an increasing number of countries exchange cotton banknotes for polymer notes is their improved counterfeit resilience^33^. Individual DFB lasers can be identified by their reflection from a white light source and the flexible nature of the membrane laser allowed repeated flexing and bending without delamination or damage. Upon pulsed excitation of the section of the banknote containing the membrane, laser action was readily observed (Fig. 3d). The lasing threshold was 38.2 kW cm^−2^, larger than for the free-standing membrane. We attribute this increased threshold to a change in waveguiding properties, with the polymer banknote acting as a substrate that reduced mode confinement and introduced leaky substrate modes.

The flexible nature of our membrane lasers also allowed reversible tuning of the emission wavelength by gradual bending parallel to the grating grooves (Supplementary Fig. 4). When the membrane was straightened out again, the original laser wavelength was accurately restored (standard deviation of laser wavelength over 20 bending cycles with ~8 mm radius of curvature, *σ*(*λ*_max_) = 50.7 pm; Fig. 3e).

To study the stability of our devices further, we repeatedly recorded the lasing spectrum of a banknote containing a membrane with three different gratings over the course of several months (Fig. 3f, Supplementary Fig. 5). During this test, the banknote was stored under ambient conditions and no encapsulation was employed. Compared to the free-standing membrane lasers (Fig. 2c, Supplementary Fig. 3), the maximum spectral linewidth of the laser emission from the banknote increased to Δ*λ*_max_ = 1.2 nm (FWHM, Supplementary Fig. 5a). However, due to the large signal-to-background ratio of the lasing spectra, the spectral position of the lasing peak can be localized to a much higher precision using peak fitting. Using Gaussian fits to the lasing spectra, we find that the standard deviation of the lasing wavelengths varies by *σ*(*λ*_max_) < 65 pm over the entire test period (Supplementary Fig. 5b). Hence, we conclude that the desired 1 nm precision needed to obtain 10^15^ unique labels is readily achievable.

### Membrane lasers as wearable security tag

**Fig. 4:**
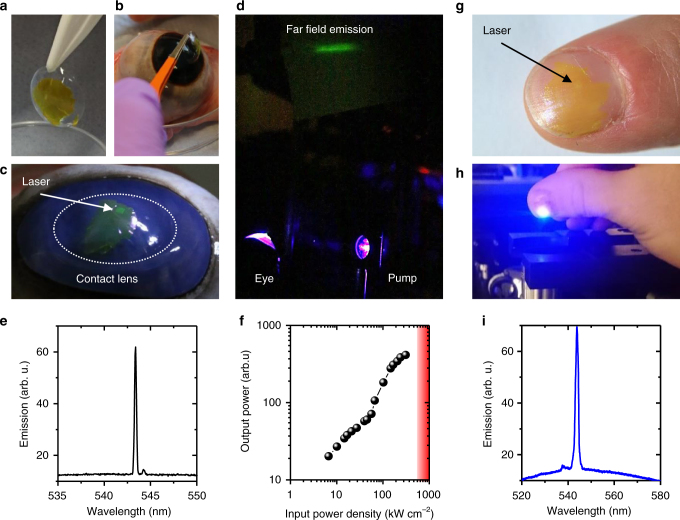
Membrane lasers as wearable security tags. **a** Membrane laser transferred onto a contact lens. **b** Contact lens laser being placed on a bovine eye. **c** Reflection of a white light source from a second-order membrane laser on bovine eye (white dashed line: outline of the contact lens; white arrow: position of grating). **d** Photograph of laser beam emitted by same bovine eye with contact lens laser, viewed on a screen placed ~50 cm away. The laser is optically pumped with blue light from the right. **e** Emission spectrum recorded from a bovine eye with contact lens containing a laser membrane. **f** Input−output characteristics of a mixed-order distributed feedback (DFB) laser on a bovine eye ball. The red area marks pump power densities that exceed the safety limit for intentional and repeated use on the human eye. **g** Membrane laser transferred onto a finger nail. **h** Optical excitation of a membrane laser on a finger nail. **i** Emission spectrum recorded from the same laser

The high optical transparency of the membrane lasers, combined with their low thresholds and ultrathin design, also inspired us to explore their use as a wearable security tag on contact lenses where they may complement a biometric authentication via an iris scan. Post lift-off, we transferred membrane lasers onto commercially available contact lenses (Fig. 4a) and mounted these on an explanted bovine eyeball (Fig. 4b, c). The bovine eye is an excellent and widely used model for the human eye due to its similar structure, slightly larger size, and general availability^34^. Upon optical excitation with pulsed blue light, we observed a well-defined green laser beam emerging from the eye (Fig. 4d). The modest divergence of the beam in the far field pattern is consistent with expectations for the second-order DFB laser used for this experiment. Mounting the membrane laser on the eye did not impede narrow linewidth, single-mode operation (peak wavelength, *λ* = 543.4 nm; Fig. 4e). Lasing action was again also confirmed by the superlinear relation between pump fluence and output power (Fig. 4f). The threshold pump fluence to achieve lasing was 56.8 kW cm^−2^, i.e. higher than the free-standing membrane laser, which we again attribute to the contact lens acting as a substrate that weakens mode confinement. Importantly, however, the power density required to operate the membrane laser is well within the maximum permissible exposure for intentional and repeated ocular exposure (ANSI 2000)^35^. For a divergent pump beam with a full visual angle of *α* = 50°, a wavelength of *λ* = 450 nm, a pulse duration of 5 ns and a repetition rate of 5 Hz, the maximum permissible corneal irradiance (thermal limit) is 505.1 kW cm^−2^ (red area in Fig. 4f), i.e. almost one order of magnitude higher than the pump power density required to operate our laser. According to the ANSI 2000 standard, a membrane laser on a contact lens could thus—under appropriate pumping conditions—be safely operated while being worn in the eye.

To further illustrate the transferability and possibility for bio-integration, we also placed a membrane laser onto a finger nail (Fig. 4g) where the spectrum can again be readily read out (Fig. 4h, i), thus providing a possible augmentation for biometric finger print scans.

## Discussion

In summary, we have demonstrated the fabrication and operation of ultra-thin, substrate-less organic lasers with extreme mechanical flexibility and lightweight. These physical properties combined with the low lasing threshold and the ability to generate unique output spectra allows the application of membrane lasers as security label that can be applied to a wide range of substrates including banknotes, contact lenses, and finger nails. In the future, the effective gain spectrum of the membrane laser can be broadened further by combining several organic polymers, which would enable a further exponential increase in the number of unique output spectra that can be generated, to (10^15^)^*n*^ for *n* different organic polymers. Further optimization of the DFB grating will likely allow lower lasing thresholds and facilitate LED pumping of membrane lasers. By combining recently developed roll-to-roll nanoimprint and organic ink jet printing technology^36^, membrane lasers could be mass-produced with high reproducibility and at low cost.

## Methods

### Membrane fabrication

A ~50 nm-thick sacrificial layer of PEDOT:PSS (Clevios P VP AI 4083, Heraeus) was spin-coated onto an oxygen plasma-treated glass substrate (25 mm × 25 mm) at 4000 rpm for 60 s and baked for 10 min at 100 °C. Subsequently, a thin (<10 nm) adhesion promoter (mr-APS1, Micro Resist Technology) and the photo-curable nanoimprint lithography resist (mr-UVCur21-200nm, Micro Resist Technology) were spin-coated and baked according to the manufacturer’s guidelines (in brief, mr-APS1 was spin-coated at 4000 rpm for 60 s and baked for 60 s at 150 °C and UVCur21-200nm was spin-coated at 3000 rpm for 60 s and baked for 20 s at 100 °C). A transparent perfluoropolyether soft stamp—comprising a negative of the final grating structure (mixed- and second-order with nominal grating periods of 340 nm, 350 nm and 360 nm, in second-order)—was molded into the UV curable polymer layer using a UV imprint alignment system (EVG620, EV Group; *λ* = 365 nm; dose 56 mW cm^−2^; exposure time 220 s). After removing the soft stamp, the grating surface (average thickness, 50 nm) was treated with an oxygen plasma to remove any remaining organic residues and reduce hydrophobicity. Three different gain materials were tested in this study: poly(9,9-dioctylfluorene-co-benzothiadiazole) with a 9:1 ratio of F8 to BT units (F8_0.9_BT_0.1_; *M*_w_ = 52,000 g mol^−1^; ADS233YE, American Dye Source Inc.), poly(9,9-dioctylfluorene-co-benzothiadiazole) with a 1:1 ratio of F8 to BT units (F8BT; *M*_w_ = 61,000 g mol^−1^; ADS133YE, American Dye Source Inc.) and a poly(para-phenylene-vinylene) copolymer (Super Yellow; *M*_w_ = 1.7×10^6^ g mol^−1^; PDY-132, Merck). These were dissolved in toluene at 25 mg ml^−1^ (F8_0.9_BT_0.1_ and F8BT) and 10 mg ml^−1^ (Super Yellow), respectively, and the resulting solutions spin-coated at 2000 rpm for 60 s, yielding a gain layer of (180 ± 10) nm (for F8_0.9_BT_0.1_ and F8BT; see Supplementary Fig. 6 for concentration-dependent layer thickness) and (250 ± 10) nm (Super Yellow). To release the membrane from the rigid glass substrate, the sample was immersed in deionized water heated to 55 °C for 1 h.

The thickness of the different layers was measured using a profilometer (Dektak 150 Surface Profilometer; Veeco). The DFB grating properties were checked using an AFM. Each DFB grating covered an area of 1 mm × 1 mm.

### Optical characterization

Membrane lasers were investigated on a custom-built inverted fluorescence microscope. The pulses produced by an optical parametric oscillator (OPO; Opolette 355, Opotek Inc.) tuned to 450 nm (repetition rate, 5 Hz; pulse duration, 5 ns) were passed through a dichroic beam splitter (cut-on wavelength, 500 nm) and focused onto the membrane lasers with a 40× or 10× objective. The emission from the membrane was collected with the same objective and passed into the collection path via the dichroic beam splitter. Bright field images were recorded with a CCD camera. To record the spectrally resolved laser emission, the light was focused onto the entrance slit of a spectrograph (Shamrock 500i, Andor) and recorded with an attached EM-CCD camera (Newton 971, Andor). The minimum peak width that can be resolved with this system is 50 pm. The pump spot size varied from 35 to 400 μm diameter.

For far field emission measurements, the back focal plane of the objective was imaged onto the entrance slit of the spectrometer by inserting an additional lens in the emission path one focal length away from the back focal plane of the objective and one focal length away from the projection lens in front of the spectrograph. To resolve the far-field pattern along both axes, the spectrograph was operated in zero-order reflection, i.e. without any wavelength dispersion.

To control the pump power density of single pulses and record the input−output characteristics of the membrane lasers, the OPO emission was passed through a pair of angle-adjustable birefringent polarizers with the angle between the polarization of the OPO emission and the first polarizer adjusted by a computer-controlled stepper motor. Emission spectra at different pump power densities were recorded and spectrally integrated to determine the laser output power.

The PLQY measurements (Supplementary Table 1) were performed in an integrating sphere, using a Hamamatsu Photonics C9920-02 measurement system. The excitation wavelength was tuned to 450 nm.

### Data availability

The datasets supporting this publication can be accessed via the PURE repository at 10.17630/be25b3ca-49e0-421f-9fb2-8f75f4bb4202.

## Electronic supplementary material

Supplementary Information
