# Supplementary material for: Flexible and ultra-lightweight polymer membrane lasers
Source: Nat Commun. 2018 May 1;9:1525. doi: 10.1038/s41467-018-03874-w (PMC5931618; doi:10.1038/s41467-018-03874-w)
Supplement: Supplementary file 1 — Supplementary Information [file 41467_2018_3874_MOESM1_ESM.pdf]

Supplementary Information:
Flexible and Ultra-lightweight Polymer Membrane Lasers

Markus Karl, James M. E. Glackin, Marcel Schubert, Nils M. Kronenberg, Graham A. Turnbull, Ifor D. W. Samuel and Malte C. Gather

Organic Semiconductor Centre, SUPA, School of Physics and Astronomy,
University of St Andrews, St Andrews KY16 9SS, United Kingdom

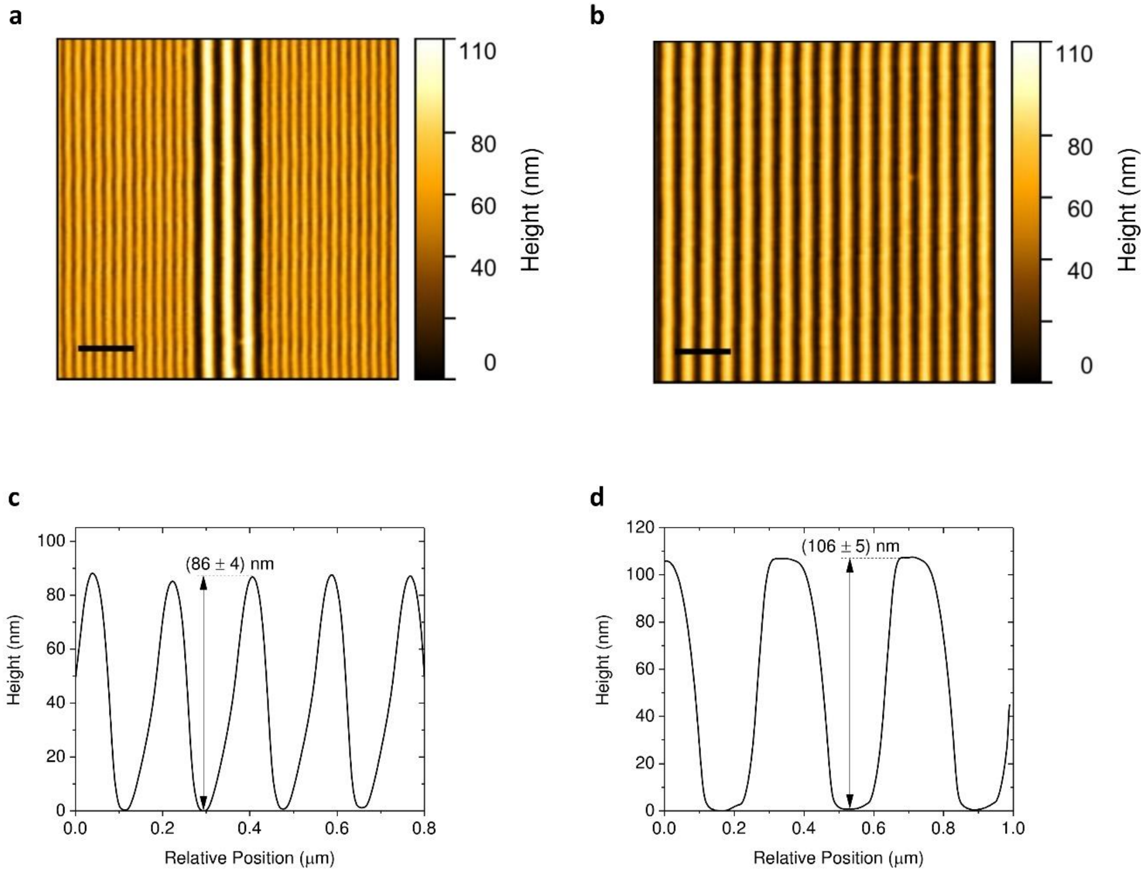

Supplementary Figure 1 | Grating characterization. a, Atomic force microscopy (AFM) measurement of a mixed-order neat UVCur grating with four second-order periods for light extraction. Scale bar, 1 μm. **b**, AFM measurement of a second-order neat UVCur grating. Scale bar, 1 μm. **c**, AFM line scan across a first-order grating region. **d**, AFM line scan across a second-order grating region.

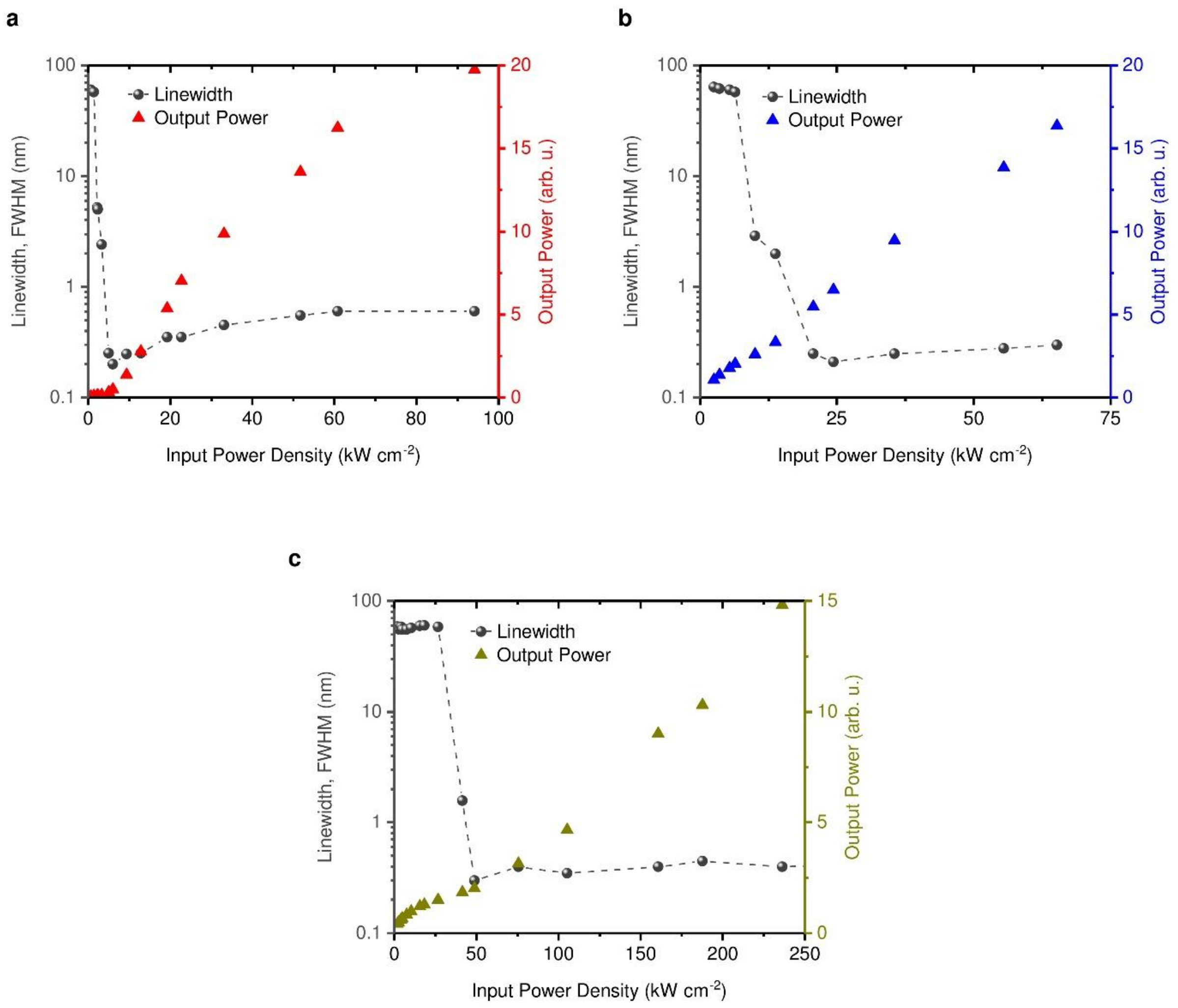

Supplementary Figure 2 | Linewidth and output power versus input power density for mixed-order membrane lasers based on different materials. Spectral linewidth (full width at half maximum, FWHM, log scale, left axis) and output power (linear scale, right axis) over input power density for lasers based on **a**, $F8_{0.9}BT_{0.1}$, **b**, F8BT and **c** Super Yellow. At low input power densities, the rather broad linewidth ($\sim 60\ nm$) originated from the fluorescence emission. For higher pump power densities, a Bragg mode with a width of $\sim 2-4\ nm$ appeared on top of the fluorescence peak. Increasing the pump power density further, led to a line narrowing to $\sim 0.2-0.4\ nm$, which is further evidence for lasing action. The super linear gain in output power above threshold is less visible for F8BT based devices due to their strong fluorescence signal below threshold.

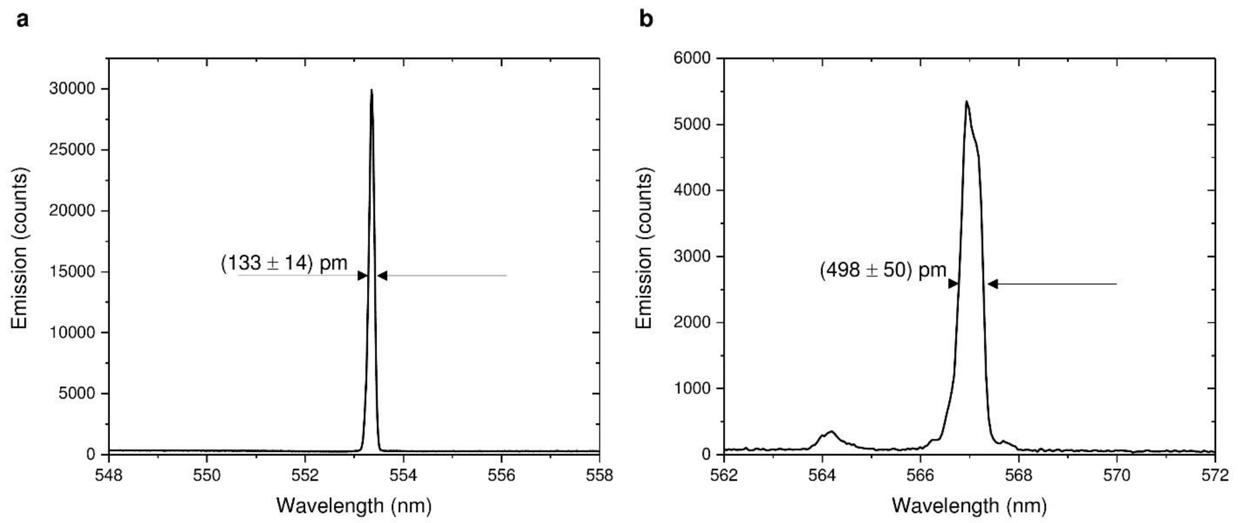

Supplementary Figure 3 | Emission linewidth of membrane laser. a, Emission spectrum of a membrane laser with a second order DFB grating. **b**, Emission spectrum of a membrane laser with a mixed order DFB grating. Linewidth at full width half maximum (FWHM) is indicated by two arrows in each panel. The pump power density for both measurements was adjusted to $120\ kW\ cm^{-2}$.

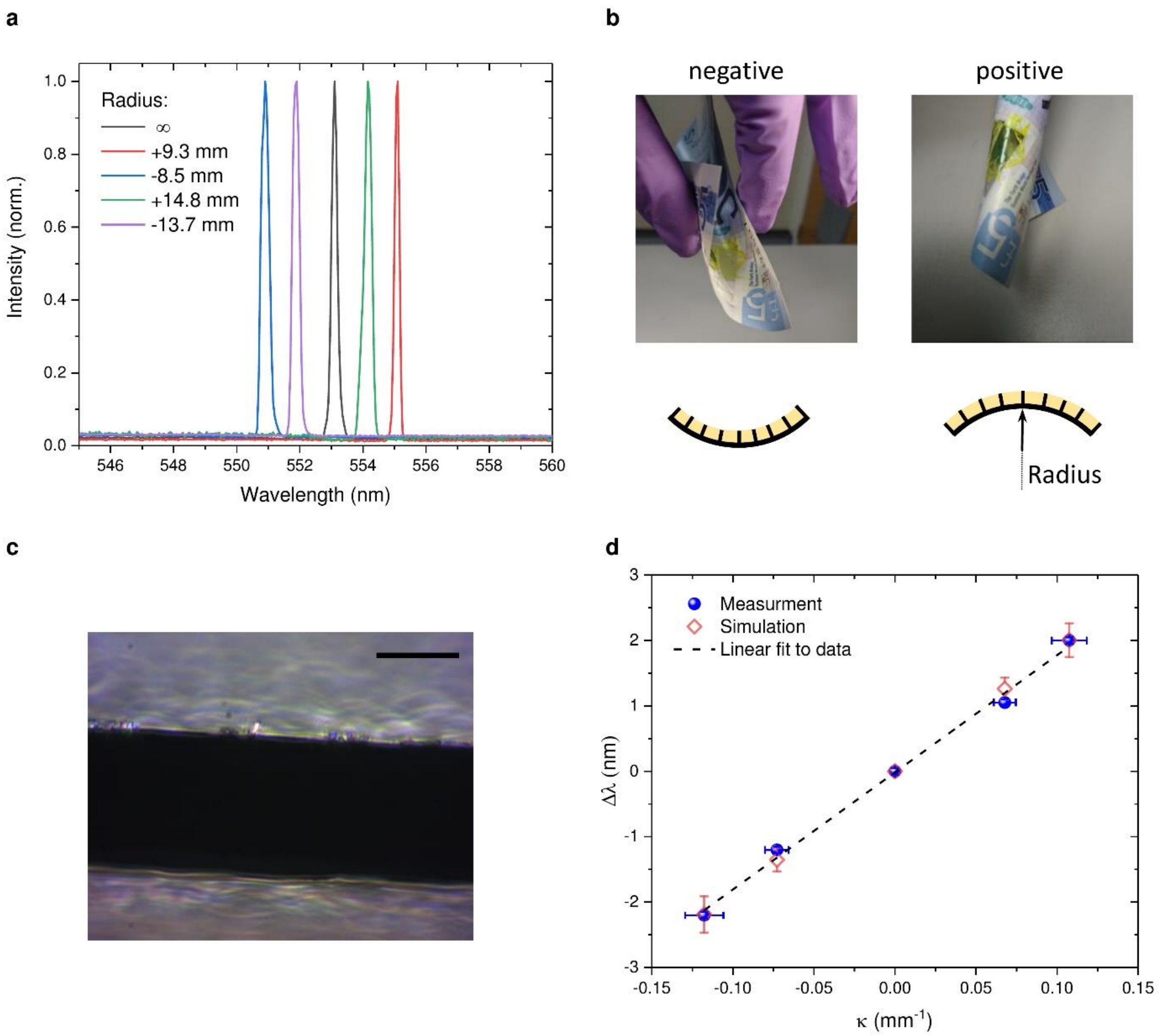

Supplementary Figure 4 | Mechanical flexibility of membrane lasers on a polymer banknote. a, Emission spectrum of a second-order membrane laser on a banknote for various bending radii. Bending applied perpendicular to the grating grooves. **b**, Photographs and illustrations showing the negative and positive bending radius of the membrane laser on a banknote. **c**, Microscopy image of the edge of the banknote (black). Measured total thickness $(84.1 \pm 6.1)\ \mu m$. Scale bar, 50 μm. **d**, Wavelength shift $\Delta\lambda$ versus bending curvature κ (inverse of radius). Blue circles indicate the measured wavelength shift extracted from a. Red diamonds represent the wavelength shift expected by calculating the grating groove displacement on top of the banknote substrate (using the thickness from c). The black dashed line is a linear fit ($R^2 = 0.996$) to the measured data. The bending sensitivity of the lasing wavelength is $(17.92 \pm 0.69) nm \times mm$.

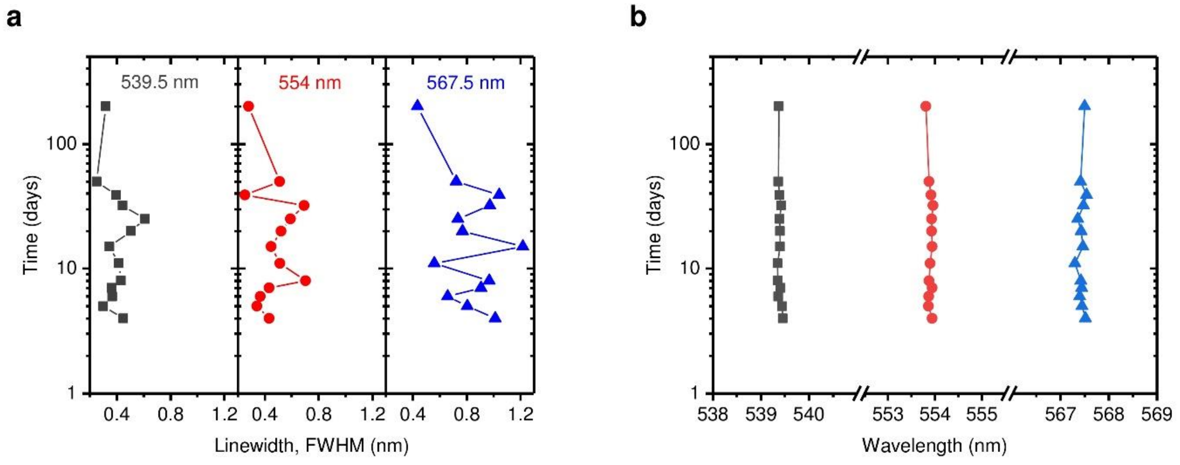

Supplementary Figure 5 | Linewidths and peak lasing wavelengths of a membrane laser on a polymer banknote over 200 days. The spectra from Fig. 3f were fitted with Gaussian functions to accurately determine the spectral linewidth (full width at half maximum, FWHM, **a**) and the peak emission wavelength (**b**). The broadest linewidth ($\Delta\lambda = 1.2\ nm$) was obtained for emission around $567.5\ nm$. We attribute the fluctuations in linewidth to differences in optical excitation (e.g. variations in the pump spot position and pump power density, $(100 \pm 30)\ kW\ cm^{-2}$) between consecutive measurements. The standard deviation of the peak wavelength was $\sigma_{539.5} = 33.4\ pm, \sigma_{554} = 43.6\ pm\ and\ \sigma_{567.5} = 64.6\ pm$ for the lasing peaks at around 539.5 nm, 554 nm and 567.5 nm, respectively.

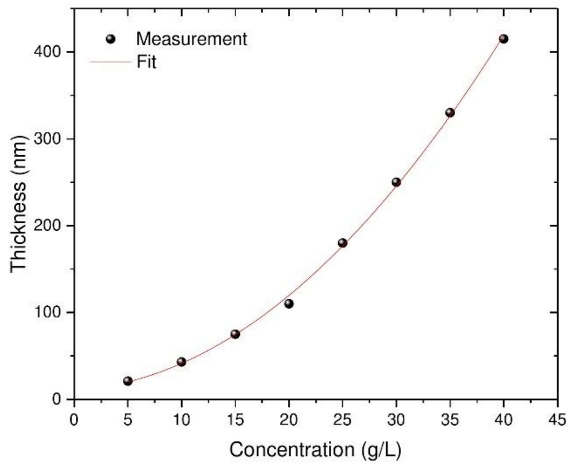

Supplementary Figure 6 | Profilometer measurement of the layer thickness of $F8_{0.9}BT_{0.1}$. Films were obtained by spin coating from toluene solutions with different concentrations (spin speed, 2000 rpm). The red line is a fit with a quadratic function.

Sample	PLQY – Pre water bath	PLQY – Post water bath
F8BT-film (neat)	0.62	0.63
F8BT membrane laser	0.33	0.57
F8BT reference	0.66	0.65
SY-film (neat)	0.43	0.38
SY membrane laser	0.31	0.30
SY reference	0.33	0.29

Supplementary Table 1 | Photoluminescence Quantum Yield (PLQY) measurement of $F8_{0.9}BT_{0.1}$ (F8BT) and Super Yellow (SY). PLQY measurements for neat films and membrane lasers (comprised of a glass substrate / PEDOT:PSS / UVCur / SY or F8BT) before and after a 1 h immersion in a water bath heated to 55 °C. The reference sample was not immersed in water but instead stored under ambient conditions for 1 h. All PLQY measurements were preformed using an integrating sphere and with an excitation wavelength of 450 nm (see Methods). The first measurement was conducted directly after spin-coating the devices. For the second measurement, the membrane laser was transferred onto a glass substrate after completing the lift-off. In neither sample, a significant reduction in PLQY due to water immersion was observed. The lower PLQY of the pre water bath laser stacks is attributed to increased absorption losses in the integrating sphere introduced by the additional PEDOT:PSS layer in the laser stack. The PEDOT:PSS layer was only present in the laser stack pre water bath where it introduced additional absorption losses that are absent in the neat film and the reference. The PEDOT:PSS layer was dissolved in the water bath, leading to an overall reduction in absorption and thus recovery in PLQY.
